# High thermal tolerance of a rainbow trout population near its southern range limit suggests local thermal adjustment

**DOI:** 10.1093/conphys/cow057

**Published:** 2016-12-09

**Authors:** Christine E. Verhille, Karl K. English, Dennis E. Cocherell, Anthony P. Farrell, Nann A. Fangue

**Affiliations:** 1 Department of Wildlife, Fish and Conservation Biology, University of California Davis, Davis, CA 95616, USA; 2 LGL Limited, Sidney, British Columbia, CanadaV8L 3Y8; 3 Department of Zoology and Faculty of Land and Food Systems, University of British Columbia, Vancouver, British Columbia, CanadaV6T 1Z4

**Keywords:** aerobic scope, fish, metabolic rate, *Oncorhynchus mykiss*, swimming, temperature

## Abstract

Transformation of earth's ecosystems by anthropogenic climate change is predicted for the 21st century. In many regions, the associated increase in environmental temperatures and reduced precipitation will have direct effects on the physiological performance of terrestrial and aquatic ectotherms and have already threatened fish biodiversity and important fisheries. The threat of elevated environmental temperatures is particularly salient for members of the *Oncorhynchus* genus living in California, which is the southern limit of their range. Here, we report the first assessments of the aerobic capacity of a Californian population of wild *Oncorhynchus mykiss* Walbaum in relationship to water temperature. Our field measurements revealed that wild *O. mykiss* from the lower Tuolumne River, California maintained 95% of their peak aerobic scope across an impressive temperature range (17.8–24.6°C). The thermal range for peak performance corresponds to local high river temperatures, but represents an unusually high temperature tolerance compared with conspecifics and congeneric species from northern latitudes. This high thermal tolerance suggests that *O. mykiss* at the southern limit of their indigenous distribution may be locally adjusted relative to more northern populations. From fisheries management and conservation perspectives, these findings challenge the use of a single thermal criterion to regulate the habitat of the *O. mykiss* species along the entirety of its distribution range.

## Abstract (precis)

California is the southern limit for indigenous rainbow trout. We studied wild-caught, Endangered Species Act (ESA)-listed fish beside their home stream and showed that the thermal aerobic performance capacity of these fish remains at 95% of peak aerobic scope across temperatures of 17.8–24.6°C. This range represents an unusually high temperature tolerance compared with conspecifics and congeneric species from northern latitudes.

## Introduction

Rainbow trout (*Oncorhynchus mykiss* Walbaum 1792) is regarded as a cold-water fish species with an indigenous range stretching across an immense temperature gradient, from the subarctic climate region of the Bering Sea to the Mediterranean climate region of Northern Baja California (Reyes, 2008). Despite this large temperature gradient and distribution range, the optimal temperature range for wild *O. mykiss* aerobic performance capacity has been determined only for indigenous populations inhabiting temperate climates. Local adaptation of thermal performance exists within the teleosts (Angilletta, 2009), but has never been shown for wild *O. mykiss* populations across their native range. Without knowledge of the variation in thermal performance among populations of *O. mykiss*, fish conservation managers apply regulatory water temperature criteria derived for higher latitude populations of *O. mykiss* for protection of lower latitude populations.

The present study considered the thermal performance of a population of *O. mykiss* located in a river near the southern limits of its native range and was prompted by a number of recent events. Foremost, global indicators show that 2014 and 2015 were the warmest years on record for the earth's climate (Blunden and Arndt, 2015; NOAA National Centers for Environmental Information, 2016). Animal populations, such as Californian *O. mykiss*, which exist at the latitudinal extremes of their biogeographical range, are expected to experience the most profound negative effects of such climate changes (Lassalle and Rochard, 2009). Second, for a fish that tends to favour pristine, cold water in most of its native habitat, native *O. mykiss* populations inhabiting the extremely warm summer temperatures of Californian rivers are evidence of considerable phenotypic plasticity (or genetic variability) within the species, allowing acclimation (or adaptation) to much warmer environmental temperature regimes. Indeed, severe thermal exposures in southern Western Australia have produced a line of introduced, hatchery-reared *O. mykiss* (Morrissy, 1973; Molony, 2001; Molony *et al*., 2004) that swim and feed at 26°C (Michael Snow, Department of Fisheries, Government of Western Australia, personal communication) and retain 50% of their peak aerobic capacity at 25°C (Chen *et al*., 2015). Interestingly, the founder population for this thermally tolerant hatchery strain was transplanted from California during the last century for recreational fisheries. Thus, with climate change continuing to shift baseline river water quality and availability (Sousa *et al*., 2011; Swain *et al*., 2014), especially in central California, where the intensification of weather extremes is triggering water crises and extreme droughts (Dettinger and Cayan, 2014), knowledge of the local thermal requirements of vulnerable key fish species becomes ever more pressing (Moyle *et al*., 2011).

Fish can adjust to warmer habitat temperatures by relocating to a cooler refuge (if available), thermally acclimating or thermally adapting (Farrell and Franklin, 2016); responses that all operate at different time scales. Indeed, the suggestion that fish might tailor their metabolic rate to habitat temperature has a long and strong history across a wide range of aquatic habitats and species (Fry, 1947, 1971; Brett and Groves, 1979; Elliott, 1982; Jobling, 1994; Hochachka and Somero, 2002; Donelson *et al*., 2012). In fact, local thermal adaptation has been thoroughly characterized for other fish species, such as stickleback populations (Barrett *et al*., 2011), temperate killifish (Fangue *et al*., 2006) and tropical killifish (McKenzie *et al*., 2013). Even within the genus *Oncorhynchus*, Fraser River watershed populations of sockeye salmon (*O. nerka* Walbaum 1792) have apparently tuned their thermal performance to meet the energetic needs of their once-in-a-lifetime upstream migration (Farrell *et al*., 2008; Eliason *et al*., 2011, 2013). The ability of *O. mykiss* to acclimate thermally is well documented (Myrick and Cech, 2000), and there appears to be the genetic potential for thermal adaptation given the successful selective breeding of *O. mykiss* lines that perform well at high temperatures (Australian lines, Molony *et al*., 2004; Japanese lines, Ineno *et al*., 2005). Nevertheless, assessments of the aerobic capacity in relation to water temperature of wild *O. mykiss* at the southern extent of their range in California are lacking. What is known for two Californian strains of *O. mykiss* (Eagle Lake and Mount Shasta; Myrick and Cech, 2000) is that the thermal performance curves for hatching success differ (Myrick and Cech, 2001) despite similar upper thermal tolerance values (CTmax). In addition, the Eagle Lake and Mount Shasta strains of *O. mykiss* grew fastest at different acclimation temperatures (19 and 22°C, respectively), but growth ceased at 25°C in both strains (Myrick and Cech, 2000).

The accumulating evidence for variation in thermal performance within and among Pacific salmon and rainbow trout populations seems incongruous with the criteria used by the US Environmental Protection Agency (EPA) to regulate water temperatures. The EPA uses a regulatory 7 day average of the daily water temperature maximum (7DADM) of 18°C for all juvenile *O. mykiss* over their entire native US range from southern California into Alaska (US Environmental Protection Agency, 2003). One way to bring greater insight into population-specific thermal tolerance and to take local adaptation and acclimation into consideration for regulatory purposes is to use a well-established non-lethal approach to study the thermal physiology of *O. mykiss* populations inhabiting unusually warm habitats. Therefore, we examined *O. mykiss* that inhabit the Tuolumne River below La Grange Diversion Dam, which is the most downstream habitat for *O. mykiss* in a watershed that drains ~2500 km^2^ of the Western Sierra Nevada mountain range. This river reach is characterized by a longitudinal thermal gradient, which increases from 12°C to occasionally as high as 26°C during summer warming over a ~25 km stretch of river. By measuring metabolic scope for activity (Fry, 1947), we tested the hypothesis that *O. mykiss* residing below the La Grange Diversion Dam on the Tuolumne River may be locally adapted to the summer habitat temperatures that can reach 26°C. Mechanistically, our experimental approach builds on a fish's ultimate requirement to have the capacity to supply oxygen for all activities (e.g. for foraging, digestion, growth, migration, predator avoidance and reproduction). The capacity to provide oxygen beyond basic needs is termed absolute aerobic scope (AAS), which, in field situations (e.g. Pörtner and Knust, 2007; Nilsson *et al*., 2009; Gardiner *et al*., 2010; Eliason *et al*., 2011; Rummer *et al*., 2014), can be estimated from the difference between routine metabolic rate (RMR) and maximal metabolic rate (MMR). Thus, by measuring RMR and MMR over a wide range of water temperatures, the portion of the temperature range where AAS (i.e. the capacity for aerobic activity) is maximized can be defined. Such information is lacking for wild *O. mykiss* in central California. For the present study, a temporary respirometry laboratory was built beside the Tuolumne River. This laboratory allowed wild juvenile *O. mykiss* to be tested at temperatures between 13 and 25°C before they were returned to their original habitat within 24 h, as required by the experimental permits.

This study has implications beyond the thermal needs for resident aquatic species because this segment of the Tuolumne River is part of a watershed that provides municipal water to >2.4 million residents of the San Francisco Bay Area and agricultural irrigation water to the Central Valley (Turlock Irrigation District and Modesto Irrigation District, 2011). The recent drought in central California has left reservoirs at historic lows (California Department of Water Resources, 2015) and has challenged the capacity to balance the environmental water flow needs of aquatic biota with the human requirements from this watershed for domestic, agricultural and recreational use. Juvenile *O. mykiss* living below the La Grange Diversion Dam have been observed exploiting summer Tuolumne River temperatures from 12 to 26°C over 25 river km (HDR Engineering, Inc., 2014). There are no additional cool-water inputs (except for rare summer rains), resulting in progressive warming of the water released from the Dam as it flows downstream. Establishing the optimal temperature range for aerobic performance of wild Californian *O. mykiss* will provide fish conservation managers with scientific support for temperature criteria that allow for optimization of this balance between human and fish requirements.

## Materials and methods

### Permitting restrictions that influenced the experimental design

Wild Tuolumne River *O. mykiss* were collected under National Marine Fisheries Service Section 10 permit no. 17913 and California Fish and Wildlife Scientific Collecting Permit Amendments. No distinction was made between resident (rainbow trout) and anadromous (steelhead) life-history forms. For permitting purposes, these fish are considered as ESA-listed California Central Valley steelhead, *O. mykiss*. Fish collection (up to a maximum of 50 individuals) was allowed only between river kilometer (RK) 84.0 and RK 63.6, and capture temperatures could not exceed 21.1°C. This permit allowed only two fish to be captured and tested each day, and all fish had to be returned to their original river habitat. Given that indirect fish mortality was limited to three fish, a precautionary measure included testing fish at the highest temperatures last (i.e. not randomly assigning test temperature). Additionally, the permit restricted test temperatures to ≤25°C. All experimental procedures were approved by the Institutional Animal Care and Use Committee (protocol no. 18196; the University of California Davis).

### Fish collection, transport and holding

Two wild *O. mykiss* were collected daily [a total of 44 fish; 22.4 g (SEM = 1.78, range 10.5–79.6 g) and 125.7 mm (SEM = 2.88)] from four primary locations on the Tuolumne River (Supplementary material, Fig. S1). The two fish were immediately scanned for a passive integrated transponder (PIT) tag to preclude re-testing a fish. The fish were transferred directly to a 13 litre container partly submerged in the river before being driven to the streamside field laboratory (<20 min) in insulated coolers filled with 25 litres of fresh river water. A water temperature logger (recording every 15 min; Onset Computer Corporation, USA) remained with the fish until testing was completed and the fish was returned to the river. At the field laboratory, located immediately downstream from the La Grange Diversion Dam, fish were transferred to holding tanks (300 litres) filled with flow-through Tuolumne River water (directly from the dam) that had passed through a coarse foam filter and then an 18 litre gas-equilibration column for aeration (12.5–13.6°C, >80% air saturation). Thus, field-acclimatized fish were placed into the holding tanks within 60–120 min of capture and remained there for 60–180 min before being transferred to one of two 5 litre automated swim tunnel respirometers (Loligo, Denmark). Routine and maximal metabolic rates were then measured at temperatures between 13 and 25°C (1°C increments).

### Swim tunnel respirometers

The swim tunnel respirometers received aerated Tuolumne River water from an 80 litre temperature-controlled sump that was refreshed every 80–90 min. Water temperature was regulated within ±0.5°C of the test temperature by passing sump water through a 9500 BTU Heat Pump (Model DSHP-7, Aqua Logic Delta Star, USA) with a high-volume pump (model SHE1.7, Sweetwater^®^, USA). Additionally, two proportional temperature controllers (model 72, YSI, USA) each ran an 800 W titanium heater (model TH-0800, Finnex, USA) located in the sump. The water temperature in the swim tunnels was monitored with a temperature probe connected through a four-channel Witrox oxygen meter (Loligo). All temperature-measuring devices were calibrated bi-weekly to ±0.1°C of a National Institute of Standards and Technology certified glass thermometer. Ammonia build-up was prevented by zeolite in the sump, which was replaced weekly. Water oxygen saturation in each swim tunnel was monitored continuously using a dipping probe mini oxygen sensor connected to AutoResp software (Loligo) through the Witrox system (Loligo). Video cameras with infrared lighting (Q-See, QSC1352W, China) continuously recorded (Panasonic HDMI DVD-R, DMR-EA18K, Japan) fish behaviour in the swim tunnels, which were shaded by black cloth to limit fish disturbance. A variable frequency drive motor generated laminar water flow through the swimming section (calibrated using a digital anemometer with a 30 mm vane wheel flow probe; Hönzsch, Germany) in each swim tunnel.

### Metabolic rate measurement

Routine and active metabolic rates of fish in the swim tunnel respirometers were measured using intermittent respirometry (Steffensen, 1989; Cech, 1990; Chabot *et al*., 2016; Svendsen *et al*., 2016). The swim tunnel was automatically sealed during measurements and flushed with fresh, aerated sump water between measurements (AutoResp software and a DAQ-PAC-WF4 automated respirometry system, Loligo). Oxygen removal from the water by the fish (in milligrams of oxygen) was measured for a minimal period of 2 min when the swim tunnel was sealed, without oxygen levels falling below 80% air saturation. No background oxygen consumption was detected without fish (performed at the end of each day with both swim tunnels; Rodgers *et al*., 2016) even at the highest test temperature (25°C). Each oxygen probe was calibrated weekly at the test temperatures using 100% (aerated distilled water) and 0% (150 ml distilled water with 3 g dissolved Na_2_SO_3_) air-saturated water.

Oxygen uptake was calculated according to the following formula:

$$\begin{array}{c} \mathrm{Oxygen uptake}\left({\mathrm{in mg O}}_{2}{\mathrm{kg}}^{-0.95}\min^{-1}\right)\\ =\left\{\left[\left(O_{2}\left(t_{1}\right)-O_{2}\left(t_{2}\right)\right)\times V\right]\;\times \;M^{-0.95}\right\}\;\times \;T^{-1}, \end{array}$$

where O_2_(*t*_1_) is the oxygen concentration in the swim tunnel at the beginning of the seal (in milligrams of oxygen per litre); O_2_(*t*_2_) is the oxygen concentration in the tunnel at the end of the seal (in milligrams of oxygen per litre); *V* is the volume of the swim tunnel (in litres); *M* is the mass of the fish (in killograms); and *T* is the duration of the measurement (in minutes). Allometric correction for variable body mass used the exponent 0.95, which is halfway between the life-stage-independent exponent determined for resting (0.97) and active (0.93) zebrafish (Lucas *et al*., 2014).

### Experimental protocol

Fish were introduced between 13.00 and 16.00 h each day into a swim tunnel at 13 ± 0.3°C, which was close to the river temperature at which most fish were caught, and left for 60 min before a 60 min training swim (Jain *et al*., 1997), during which water flow velocity was gradually increased to 5–10 cm s^−1^ higher than when swimming started (typically at 30 cm s^−1^) and held for 50 min before a 10 min swim at 50 cm s^−1^ (the anticipated maximal prolonged swimming velocity for a 150 mm fish at 13°C; Alsop and Wood, 1997). Recovery for 60 min preceded the incremental increases in water temperature (1°C per 30 min) up to the test temperature. Oxygen uptake (10–30 min, depending on the test temperature, and followed by a 5–10 min flush period) was continuously measured throughout the night until 07.00 h. Estimates of RMR for each of the 44 tested fish were calculated by averaging the lowest four oxygen uptake measurements at the test temperature for the minimum 8 h overnight period (Chabot *et al*., 2016). Visual inspection of the video recordings confirmed that fish were quiescent during these measurements with the exception of three fish that were discarded owing to consistent activity throughout the night (Crocker and Cech, 1997), which reduced the RMR measurements to 41 fish.

Critical swimming velocity and burst swimming protocols (Reidy *et al*., 1995; Killen *et al*., 2007; Clark *et al*., 2013; Norin and Clark, 2016) were used to determine MMR. They began between 08.00 and 09.00 h and lasted 2–6 h. For the critical swimming velocity test, water velocity was gradually increased until the fish continuously swam at 30 cm s^−1^ for 20 min. Water velocity was incrementally increased every 20 min by 10% of the previous test velocity (3–6 cm s^−1^) until the fish was no longer able to swim continuously and fell back to make full body contact with the downstream screen of the swimming chamber. The fish recovered for 1 min at 13–17 cm s^−1^, the lowest velocity setting of the swim tunnel, before restoring the final water velocity over a 2 min period and restarting the 20 min timer. Fatigue was defined as when the fish made full body contact with the downstream screen of the swim tunnel a second time at the same test velocity or failed to resume swimming. Active metabolic rate was measured at each test velocity using a 3 min flush period and a 7–17 min measurement period. All fish swam for 20 min at one water velocity, but almost 50% of the wild fish used their caudal fin to prop themselves on the downstream screen of the swim tunnel to avoid swimming faster, which required a secondary measurement of maximal metabolic rate using a burst swimming protocol. For the burst swimming protocol, tunnel velocity was set to and held for 10 min at the highest critical swimming velocity test increment where that fish had continuously swum. Afterwards, water velocity was rapidly (over 10 s) increased to 70–100 cm s^−1^, which invariably elicited burst swimming activity for 30 s or less, when water velocity exceeded 70 cm s^−1^. This protocol was repeated multiple times for 5–10 min, while oxygen uptake was measured continuously. The MMR was assigned to the highest active metabolic rate measured with the active respirometry methods. Occasionally, fish exhibited intense struggling behaviours with an even higher oxygen uptake, which was assigned MMR. The MMR was not estimated for four fish, which failed to swim and raise their metabolic rate appreciably with any of the methods, resulting in a total of 37 fish with RMR and MMR measurements. Absolute aerobic scope (AAS = MMR − RMR) and factorial aerobic scope (FAS = MMR/RMR) were calculated.

All fish recovered in the swim tunnel at a water velocity of 13–17 cm s^−1^ and at the test temperature for 1 h while measuring oxygen uptake. Water temperature was then decreased to ~13°C over a 30 min period before the fish was removed, measured, PIT tagged and put into a holding tank before release at the capture site. Fish were individually anaesthetized for <5 min with CO_2_ (2 Alka-Seltzer tablets dissolved in 3 litres of river water) for morphometric measurements [fork length (FL), in millimetres; and body mass, in grams], condition factor calculation (CF = body mass × 10^5^/FL^3^), and PIT tagging. Half duplex PIT (Oregon RFID) tags were placed into the abdominal cavity via a 1 mm incision through the body wall, just off-centre of the linea alba. All equipment was sterilized with NOLVASAN S prior to tagging, and incisions were sealed with 3M VetBond. Revived fish were immediately transported to the coolers filled with 13–15°C river water. At the release site, river water was gradually added to the cooler to equilibrate the fish to river water temperature at a rate of 1–2°C h^−1^ before fish were allowed to swim away voluntarily.

### Measurements of tail beat frequency

The tail beat frequency (TBF; number of tail beats per 10 s, reported in Hz) of fish swimming continuously and holding station without contacting the downstream screen of the respirometer was measured using the average of two or three 10 s sections of video recordings played back at either one-quarter or one-eighth of real time. The TBF was then related to swimming speed and temperature. Tail beat frequencies of undisturbed fish holding station in the Tuolumne River were measured from footage from underwater video cameras anchored within 1 m of *O. mykiss* schools and left to record for up to 4 h (GoPro Hero 4). The TBFs were determined using the same methodology applied to respirometer video recordings (*n* = 15 at 14°C and *n* = 1 at 20°C).

### Data analysis

A statistical model was fitted to individual data [performed in R (R Core Development Team, 2013) using the ‘lm’ function] to determine the best relationships between the test temperature and RMR, MMR, AAS and FAS. The statistical model (linear, quadratic, antilogarithic base 2 and logarithmic base 2 were tested) with the highest *r*^2^ and lowest residual SE being reported. Confidence intervals and predicted values based on the best-fit model were calculated in R using the ‘predict’ function. Variances around metabolic rate measurements are reported as 95% confidence intervals (CIs).

## Results

**Figure 1:**
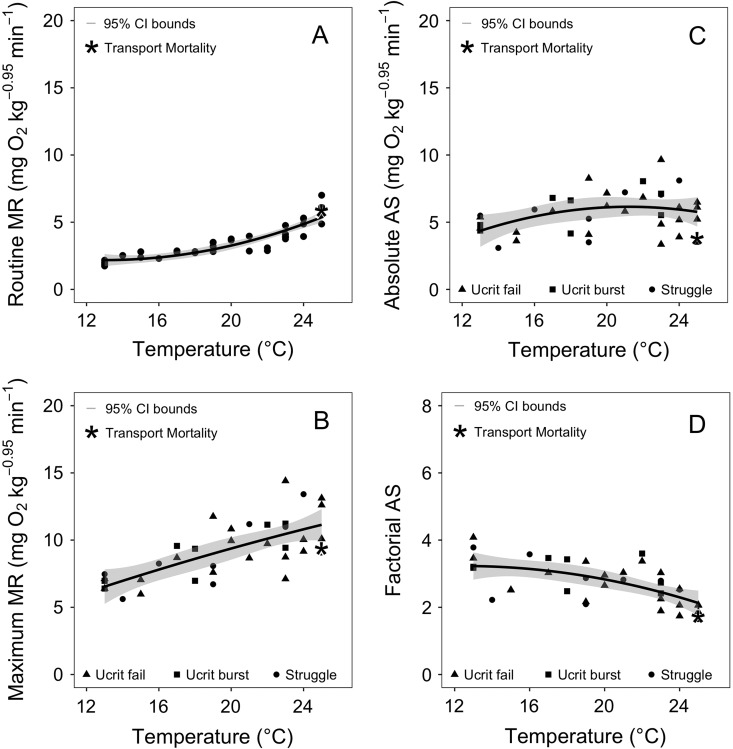
The relationships between test temperature and the routine (RMR; **A**) and maximal metabolic rate (MMR; **B**) of Tuolumne River *Oncorhynchus mykiss*. The three methods used to measure MMR (see Materials and methods section) are distinguished by different symbols. Absolute aerobic scope (AAS; **C**) and factorial aerobic scope (FAS; **D**) were derived from the metabolic rate measurements. Each data point represents an individual fish tested at one temperature. These data were given a best-fit mathematical model (continuous line or curve), and the 95% confidence intervals for each line are indicated by the shaded area. The RMR and FAS were smoothed to a polynomial fit of the form *y* = *x* + *I*(*x*^2^), where *y* is RMR or FAS, *x* is temperature, and *I* is a constant. The MMR and AAS were smoothed to a linear fit of the form *y* = *x* + *c*, where *c* is a constant. For RMR, degrees of freedom (d.f.) = 34, *P* < 0.001, residual standard error (RSE) = 0.561 and *r*^2^ = 0.798. For MMR, d.f. = 35, *P* < 0.001, RSE = 1.580 and *r*^2^ = 0.489. For AAS, d.f. = 35, *P* = 0.060, RSE = 1.490 and *r*^2^ = 0.098. For FAS, d.f. = 34, *P* < 0.001, RSE = 0.506 and *r*^2^ = 0.344. The asterisk indicates the one fish that died abruptly after the swimming test.

As anticipated, basic oxygen needs (RMR) increased exponentially by 2.5-fold from 13 to 25°C (from 2.18 ± 0.45 (95% CI) to 5.37 ± 0.41 mg O_2_ kg^−0.95^ min^−1^). This thermal response was modelled by: RMR (in mg O_2_ kg^−0.95^ min^−1^) = 5.9513 – 0.5787 (temperature, in °C) + 0.0200 (temperature, in °C)^2^ (*P* < 0.001, *r*^2^ = 0.798; Fig. 1A). The MMR increased linearly by 1.7 times (from 6.62 ± 1.03 to 11.22 ± 0.86 mg O_2_ kg^−0.95^ min^−1^) from 13 to 25°C. This thermal response was modelled by: MMR (in mg O_2_ kg^−0.95^ min^−1^) = 1.6359 + 0.3835 (temperature, in °C) (*P* < 0.001, *r*^2^ = 0.489; Fig. 1B). Given that MMR almost kept pace with the thermal effect on RMR, AAS had a rather flat reaction norm that was largely independent of the test temperature range. This thermal response was modelled by: AAS (in mg O_2_ kg^−0.95^ min^−1^) = −5.7993 + 1.1263 (temperature, in °C) − 0.0265 (temperature, in °C)^2^ (*P* = 0.060, *r*^2^ = 0.098; Fig. 1C). Using this model, peak AAS (6.15 ± 0.71 mg O_2_ kg^−0.95^ min^−1^) was centred at 21.2°C. Nevertheless, the unexpected flat reaction norm meant that 95% of peak AAS was maintained from 17.8 to 24.6°C, which is a broad thermal window for peak AAS that extends well beyond the 7DADM value of 18°C for *O. mykiss*.

Factorial aerobic scope is a useful metric of whether or not a fish might have the required aerobic capacity to perform a specific activity, e.g. a doubling of RMR (i.e. FAS = 2) might be needed to digest a full meal properly (Jobling, 1981; Alsop and Wood, 1997; Fu *et al*., 2005; Luo and Xie, 2008). As expected, FAS decreased with temperature (Clark *et al*., 2013), a thermal response modelled by: FAS = 2.1438 + 0.1744 (temperature, in °C) − 0.0070 (temperature, in °C)^2^ (*P* < 0.001, *r*^2^ = 0.344; Fig. 1D).

**Figure 2:**
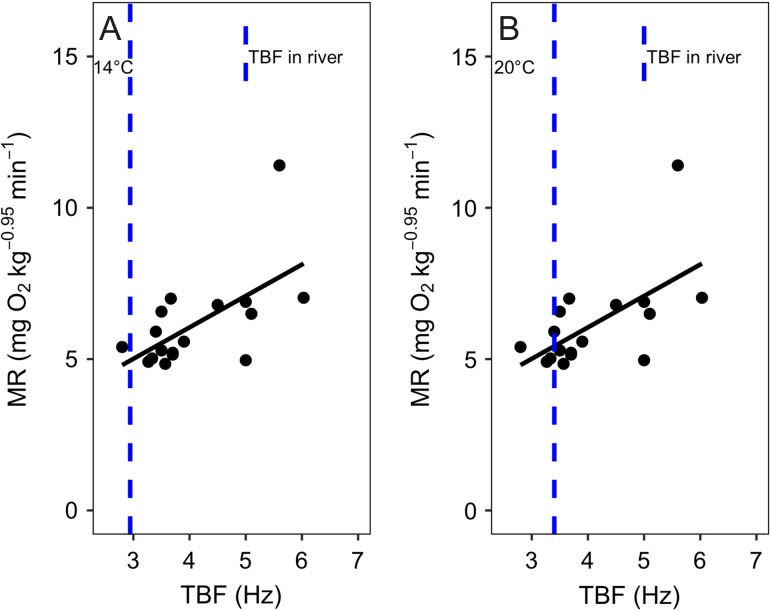
The relationship between tail beat frequency (TBF; in hertz) and metabolic rate (MR; in mg O_2_ kg^−0.95^ min^−1^) measured when Tuolumne River *Oncorhynchus mykiss* were swimming continuously in a swim tunnel at 14 (**A**) or 20°C (**B**). The continuous black line represents the linear regression based on the data for *n* = 7 fish at 14°C and *n* = 5 fish at 20°C. The vertical dashed lines represent the estimated TBF (2.94 Hz at 14°C and 3.40 Hz at 20°C) taken from videos of *O. mykiss* maintaining station in a water current in their normal Tuolumne River habitat. At 14°C, the relationship between TBF and MR followed the equation MR = 0.75TBF + 1.05, with degrees of freedom (d.f.) = 41, *P* < 0.001, residual standard error (RSE) = 1.27 and *r*^2^ = 0.35. According to this formula, the MR for the TBF measured in the river (2.943 Hz) at 14°C was estimated to be 3.26 mg O_2_ kg^−0.95^ min^−1^. At 20°C, the relationship between TBF and MR followed the equation MR = 1.04TBF + 1.89, with d.f. = 15, *P* = 0.009, RSE = 1.29 and *r*^2^ = 0.33. According to this formula, the MR for the TBF measured in the river at 20°C (3.402 Hz) was estimated to be 5.43 mg O_2_ kg^−0.95^ min^−1^.

In addition, given the need to integrate AAS or FAS within an ecological framework (see Overgaard *et al*., 2012; Clark *et al*., 2013; Farrell, 2013, 2016; Pörtner and Giomi, 2013; Ern *et al*., 2014; Norin *et al*., 2014), we used measurements of TBF to estimate the oxygen cost required by a wild *O. mykiss* to maintain station in the river currents of typical habitats in the Tuolumne River. A steady TBF used for this activity at ambient temperatures of 14 and 20°C was 2.94 and 3.40 Hz, respectively (see supplemental video, available online). Using respirometer swimming data to relate TBF to oxygen uptake at 14 and 20°C (*P* < 0.001, *r*^2^ = 0.35; and *P* = 0.009, *r*^2^ = 0.33, respectively; Fig. 2), in-river TBF values of 2.94 and 3.40 Hz corresponded to 3.26 and 5.43 mg O_2_ kg^−0.95^ min^−1^, respectively. Therefore, we suggest that wild fish observed holding station in the Tuolumne River increased RMR by 1.5 times at 14°C and by 2.0 times at 20°C, an activity that would use 49 and 67%, respectively, of the available FAS measured at these two temperatures.

Fish recovery after exhaustive swimming tests was quick and without any visible consequences. The RMR at the end of the 60 min recovery period was either elevated by no more than 20% or fully restored; an observation consistent with previous laboratory studies of *O. mykiss* recovery (Jain *et al*., 1997; Jain and Farrell, 2003). Two fish tested at 25°C were the only exceptions. These two fish regurgitated their gut contents during recovery and one then died abruptly. Inadvertent fish recapture provided some information on fish survival after being returned to the river. Six PIT-tagged fish were recaptured at 1–11 days post-testing within 20 m of their original capture location; all were visually in good condition, and three of these fish had been tested at 23°C.

## Discussion

The present study is the first to consider the thermal response for an *O. mykiss* population so close to the southerly boundary of the natural distribution range for indigenous *O. mykiss*. We clearly show that 95% of peak AAS was maintained over an unexpectedly broad thermal window (17.8–24.6°C) and that all fish tested could maintain an FAS >2.0 up to 23°C. Moreover, we place these findings into an ecological context by suggesting that the level of FAS at temperatures at least as high as 20°C may be more than adequate to maintain station in the local water current of the Tuolumne River and probably to digest a meal properly and optimize growth, which is a very powerful integrator of environmental, behavioural and physiological influences on a fish's fitness. Moreover, fish were tested on site and returned afterwards to the river, making the work locally relevant for the *O. mykiss* population, sensitive to conservation needs and globally relevant by addressing the following broad question: are fish at the extreme edges of their biogeographical range more physiologically tolerant because of the thermal extremes they experienced there?

The present results show good quantitative agreement with various previous studies with *O. mykiss* that have measured some of the variables measured in the present study. For example, the 2.5-fold exponential increase in RMR from 13 to 25°C (from 2.18 ± 0.45 (95% CI) to 5.37 ± 0.41 mg O_2_ kg^−0.95^ min^−1^) compares well with laboratory studies of RMR reported at 14°C (2.3–2.8 mg O_2_ kg^−0.95^ min^−1^; Myrick and Cech, 2000) for 7 g Mount Shasta and Eagle Lake *O. mykiss*, and at 25°C (~6.5 mg O_2_ per kg^−0.95^ min^−1^; Chen *et al*., 2015) for 30 g Western Australian *O. mykiss*. Therefore, concerns about handling stress and specific dynamic action were minimal. Likewise, MMR increased linearly by 1.7 times (from 6.62 ± 1.03 to 11.22 ± 0.86 mg O_2_ kg^−0.95^ min^−1^) from 13 to 25°C, comparing well with previous laboratory measurements of MMR reported at 15°C (2.8–8.7 mg O_2_ kg^−0.95^ min^−1^) for 2–13 g *O. mykiss* (Scarabello *et al*., 1992, Alsop and Wood, 1997) and with the peak MMR at 20°C (~11.13 mg O_2_ per kg^−0.95^ min^−1^) for Australian *O. mykiss* (Chen *et al*., 2015). As a consequence of MMR nearly keeping pace with the thermal effect on RMR, AAS was largely independent of test temperature. Directly comparing our AAS values with other studies revealed that our result for AAS at 15°C (5.10 mg O_2_ kg^−0.95^ min^−1^) was at the high end of previous laboratory measurements of AAS (1.8–5.8 mg O_2_ kg^−0.95^ min^−1^) for *O. mykiss* at 15°C (Scarabello *et al*., 1992, Alsop and Wood, 1997; McGeer *et al*., 2000), but lower than peak AAS (~7.3 mg O_2_ per kg^−0.95^ min^−1^) at 20°C in Australian *O. mykiss* (Chen *et al*., 2015). Likewise, our FAS values were bracketed by values obtained in previous laboratory studies. At 24°C, FAS (2.13 ± 0.33) was greater than that reported at 25°C (1.8) for Western Australian *O. mykiss* (Chen *et al*., 2015), but compared with FAS values for juvenile rainbow trout (1.8–5.8) at 13°C (Scarabello *et al*., 1992, Alsop and Wood, 1997, McGeer *et al*., 2000), our FAS at 13°C (3.32 ± 0.41) was in the middle of the range.

**Figure 3:**
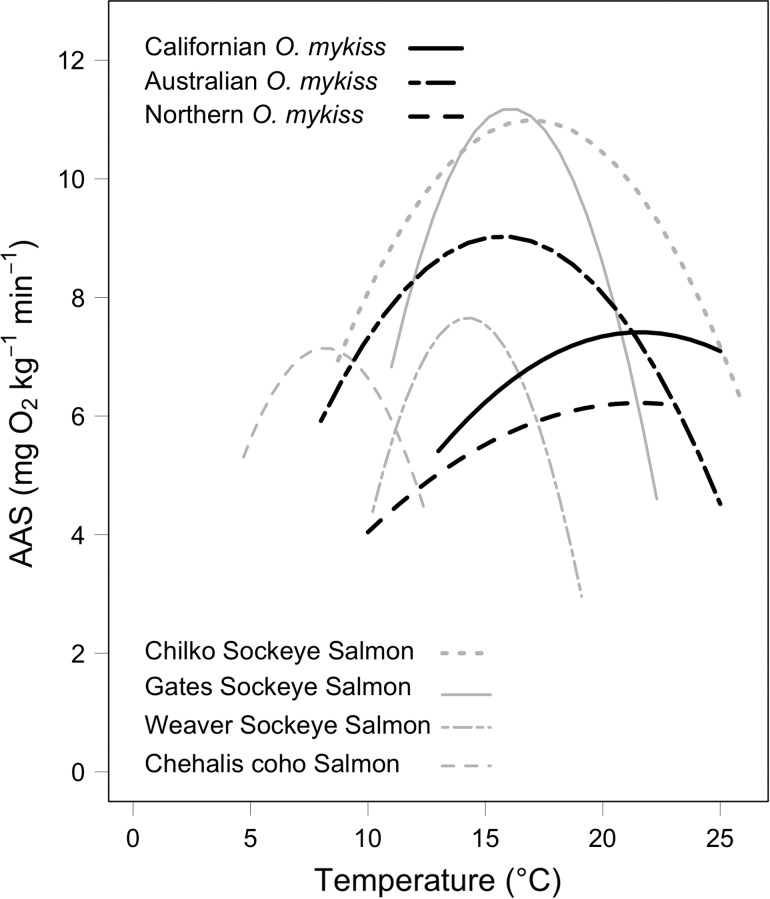
Absolute aerobic scope (AAS) for three strains of *Oncorhynchus mykiss*, i.e. a northern strain (Fry, 1948), an Australian strain (Chen *et al*., 2015) and the California strain reported in this manuscript, compared with AAS measurements of Chehalis Coho salmon (*Oncorhynchus kisutch* Walbaum 1792) and Gates Creek, Weaver Creek (*Oncorhynchus nerka* Walbaum 1792; Lee *et al*., 2003a) and Chilko Creek sockeye salmon (Eliason *et al*., 2011). The best-fit line of the relationship between AAS and temperature of the species and populations from other publications was predicted using a second-order polynomial linear regression performed on the raw data (Lee *et al*., 2003a; Chen *et al*., 2015) or data extracted from plots (Fry, 1948; Eliason *et al*., 2011) from the original publications. Coefficient estimates from the linear regression analysis were then used to determine the peak aerobic scope and the temperatures corresponding to the peak and 95% of peak AAS.

To place the present data for Californian *O. mykiss* into perspective, we have compared (Fig. 3) their reaction norm with those published for juveniles of northern *O. mykiss* (data from Fry, 1948) and Australian hatchery-selected *O. mykiss* (data from Chen *et al*., 2015), as well as adult northern populations of selected Pacific salmon populations (data from Lee *et al*., 2003a and Eliason *et al*., 2011). Among the native *O. mykiss* populations, the Lower Tuolumne River juvenile Californian *O. mykiss* are likely to experience the highest temperatures during summer (up to 26°C), although the introduced Australian *O. mykiss* population had experienced selection temperatures ≥25°C (Chen *et al*., 2015). Notably, AAS at 24°C for Tuolumne River *O. mykiss* is greater than other *O. mykiss* populations and only bettered by the Chilko sockeye salmon population, one of several sockeye salmon populations that are known to have a peak AAS at the modal temperature for their upstream spawning migration (Eliason *et al*., 2011, 2013). Thus, the present data are in line with evidence of intraspecific matching of metabolic rate to local water temperatures within the *Oncorhynchus* genus. Although the peak AAS of the Australian *O. mykiss* population was 50% greater than for the other two *O. mykiss* populations, Tuolumne River *O. mykiss* had the broadest and highest thermal window (17.8–24.6°C) among the *O. mykiss* populations (20.5–22.4°C from Fry, 1948; and 12.8– 18.6°C from Chen *et al*. 2015).

Whether the matching of Tuolumne River *O. mykiss* metabolic performance to local habitat temperatures is a result of thermal acclimation or local adaption, as in the Western Australian *O. mykiss*, will need study well beyond the present work. Thermal acclimation usually results in fish performing better at the new temperature. For example, thermal acclimation offsets the effect of acute warming on RMR in 5–7 g Mount Shasta and Eagle Lake *O. mykiss* (2.3–2.8 mg O_2_ kg^−0.95^ min^−1^ at 14°C and 2.9–3.1 mg O_2_ kg^−0.95^ min^−1^ at 25°C; Myrick and Cech, 2000), which would normally double RMR over this temperature range, as observed here. Warm acclimation can also increase upper thermal tolerance limits, as it did for four anadromous Great Lakes populations of juvenile *O. mykiss* (Bidgood and Berst, 1969). Given that our fish were captured at and, presumably, thermally acclimatized to between 14 and 17°C, it would be of interest to test wild fish with a warmer thermal acclimation history. But even without thermal acclimation, the present data suggest that Tuolumne River *O. mykiss* and those for northern *O. mykiss* (Fry, 1948; see Fig. 3) have the aerobic capacity temporarily, if not regularly, to exploit temperatures well above 18°C, which is the upper thermal limit suggested by EPA guidance documents (US Environmental Protection Agency, 2003).

Nevertheless, we caution that such local tailoring may not be evident in all salmonid species. For example, the thermal physiology of Atlantic salmon (*Salmo salar* Linnaeus 1758) from northern and southern extremes of their European range did not show any major difference (Anttila *et al*., 2014). All the same, a sub-species of redband trout (*O. mykiss gairdneri*), which are apparently adapted to high summer temperatures of North American desert streams (Narum *et al*., 2010; Narum and Campbell, 2015), are likewise capable of high levels of swimming performance up to 24°C (Rodnick *et al*., 2004) and higher swimming performance for a warm vs. a cool creek population (Gamperl *et al*., 2002). Redband trout have been observed actively feeding at 27–28°C (Sonski, 1984; Behnke, 2010), but thermal selection of wild redband trout is centred between 13 (Gamperl *et al*., 2002) and 17°C (Dauwalter *et al*., 2015). How *O. mykiss* behaviourally exploit the steep summer thermal gradient in the Tuolumne River below the La Grange Diversion Dam (from 12 to 26°C over 25 km; HDR Engineering, Inc., 2014) is another unknown. Even without these important details, Tuolumne River *O. mykiss* appear physiologically to be tolerant of the thermal extremes they experience.

The capacity of a fish to deliver oxygen to support activities in water of varying quality is a concept originally introduced for fishes >60 years ago (Fry, 1947). The oxygen- and capacity-limited thermal tolerance hypothesis broadens this concept and provides a mechanistic explanation (Pörtner, 2001; Pörtner and Farrell, 2008; Deutsch *et al*., 2015), but is currently under debate (Overgaard *et al*., 2012; Clark *et al*., 2013; Farrell, 2013; Pörtner and Giomi, 2013; Ern *et al*., 2014; Norin *et al*., 2014). An accepted fact is that a metabolic load from an environmental factor (e.g. temperature) can increase the oxygen cost for living (i.e. RMR). Consequently, like all other temperature studies with fish, the magnitude of the 2.5-fold increase in RMR observed here over a 12°C temperature range (between 13 and 25°C) was expected. However, temperature did not limit MMR, which increased linearly with acute warming, and the peak MMR was not resolved. The statistical models, which were based on individual responses and 1°C temperature increments from 13 to 25°C, predicted a peak AAS at 21.2°C for Tuolumne River *O. mykiss* and a FAS >2.0 up to 23°C. As the allocation of energy and trade-offs are recognized and fundamental tenants of ecological physiology, especially in fishes (Sokolova *et al*., 2012), we suggest that being able to at least double RMR has ecological relevance for two behaviours that are likely to influence survival of *O. mykiss*, maintaining station in a flowing river and processing a large meal.

Snorkeling in the Tuolumne River provided visual observations of *O. mykiss* maintaining station in the river current for prolonged periods that were punctuated by hiding under the river bank and by darting behaviours to capture prey and to protect their position. Maintaining station required a steady TBF similar to the situation in the swim tunnel respirometer, which allowed us to estimate a metabolic cost of maintaining station in typical Tuolumne River habitats at 14 and 20°C (a 1.5- to 2-fold increase in RMR) and the aerobic scope available for additional activities (1.7–2 times RMR). Although darting behaviours are likely to be fuelled anaerobically, *O. mykiss* must (and were clearly able to) repay the post-exercise excess oxygen debt (Lee *et al*., 2003b) while maintaining station in the river current. The rapid recovery of RMR after exhaustive exercise in the swim tunnel suggests that *O. mykiss* had the capacity to repay post-exercise excess oxygen debt rapidly at temperatures as high as 24°C. Although digestion of a meal at high temperatures proceeds more rapidly and with a higher peak metabolic rate, the total oxygen cost of the meal remains similar. Thus, fish can theoretically eat more frequently and potentially grow faster at a higher temperature provided there is a sufficient FAS for digestion within the overall AAS. Given that peak metabolic rate during digestion of a typical meal for a salmonid does not necessarily double RMR at the temperatures used here (e.g. Jobling, 1981; Alsop and Wood, 1997; Fu *et al*., 2005; Luo and Xie, 2008), an FAS value of 2 should be a reasonable index, and all *O. mykiss* tested had this capacity up to 23°C. Indeed, the fish were apparently feeding well in the river, given a high condition factor (1.1 SEM = 0.01), the faecal deposits found in the swim tunnel and two fish regurgitating meals when tested at 25°C. Meal regurgitation would be consistent with an oxygen limitation, given that aquatic hypoxia impairs digestion in *O. mykiss* (Eliason and Farrell, 2014). Indeed, feeding and growth are suppressed at supra-optimal temperatures (Hokanson *et al*., 1977; Brett and Groves, 1979; Elliott, 1982; Myrick and Cech, 2000, 2001). Taken together, these data suggest that Tuolumne River *O. mykiss* were doing well in their habitat and had the aerobic capacity to do so.

Our metabolic measurements, which show good quantitative agreement with controlled laboratory *O. mykiss* studies, represent a major challenge to the use of a single thermal criterion to regulate *O. mykiss* habitat when determining conservation criteria along the entire Pacific coast and perhaps elsewhere. The 7DADM of 18°C for *O. mykiss* draws heavily on a growth study performed in Minnesota (Hokanson *et al*., 1977). Therefore, it will be important to examine whether the peak AAS at 21.2°C for Tuolumne River *O. mykiss* is associated with a peak growth rate. In this regard, the peak growth rate of another Californian *O. mykiss* population (the Mount Shasta strain) occurred at acclimation temperatures (19–22°C; Myrick and Cech, 2000) above the 7DADM and within the thermal window for 95% peak AAS for Tuolumne River *O. mykiss.* The Mount Shasta *O. mykiss* strain also stopped growing at 25°C, the same temperature at which FAS for Tuolumne River *O. mykiss* approached 2. In contrast, the Californian Eagle Lake *O. mykiss* strain grew fastest at 19°C and lost weight at 25°C (Myrick and Cech, 2000). Thus, the Mount Shasta and Tuolumne River *O. mykiss* populations are better able to acclimate thermally to temperatures >20°C than the Eagle Lake strain. With clear evidence that a California strain of *O. mykiss* can grow faster at acclimation temperatures >18°C and that strains may differ in their the optimal temperature for growth by as much as 3°C, there is a precedent that local populations of *O. mykiss* can perform well above 18°C. Our findings also highlight the need for future experiments that consider replicate populations from throughout the species range to assess how widespread intraspecific variation in aerobic scope in *O. mykiss* might be. Continual development and refinement of the metrics used to best inform regulatory criteria should be an ongoing pursuit, particularly if regional standards are to be implemented and if the criteria move away from what may now be considered conservative. Probabilistic modelling approaches associated with a diversity of water temperature standards should be developed in order for managers to understand the balance between standards that are conservative compared with those that are more risky.

The capacity to balance the essential environmental requirements of aquatic biota with human requirements is becoming increasingly challenging across the globe because of recent increases in severe drought and record high temperature occurrences, a trend that climate change models project will continue. We suggest that broadly applying regulatory criteria, such as the 18°C 7DADM criterion for Pacific Northwest *O. mykiss* populations, to all North American *O. mykiss* is no longer realistic and, in the present case, overly conservative. The high degree of thermal plasticity discovered here for the Tuolumne River *O. mykiss* population, which corresponds to local thermal conditions, adds to the accumulating evidence of the capacity for local adaptation among populations within the *Oncorhynchus* genus, including *O. mykiss*. Importantly, this work clearly illustrates that, owing to thermal plasticity, broad application of a single temperature criterion for fish protection and conservation is not scientifically supported, especially for fish populations at the extreme limits of the species’ indigenous range.
